# Association between the red cell distribution width-to-albumin ratio and recurrence-free survival and overall survival in patients with non-muscle-invasive bladder cancer: a retrospective cohort study

**DOI:** 10.3389/fonc.2025.1710047

**Published:** 2026-01-05

**Authors:** Feifan Song, Shiqiang Su, Xueqiao Zhang, Yunpeng Cao, Xiongjie Cui, Lili Zhang, Chao Li, Shen Li

**Affiliations:** 1 Department of Urology, Shijiazhuang People’s Hospital, Shijiazhuang, China; 2 Graduate School of Hebei Medical University, Shijiazhuang, China

**Keywords:** albumin, non-muscle-invasive bladder cancer, overall survival, recurrence-free survival, red cell distribution width

## Abstract

**Background:**

The prognostic utility of the red cell distribution width-to-albumin ratio (RAR) in non-muscle-invasive bladder cancer (NMIBC) has not been established. This study aimed to evaluate the associations between preoperative RAR and both recurrence-free survival (RFS) and overall survival (OS) in patients with NMIBC.

**Methods:**

A retrospective review was performed for 240 individuals with NMIBC having undergone transurethral resection of bladder tumor (TURBT) at Shijiazhuang People’s Hospital from November 2013 to January 2024. Demographic characteristics, hematological parameters, pathological data, and information on instillation therapy were collected. The optimal RAR cutoff was identified by applying receiver operating characteristic (ROC) analysis. Survival curves were generated via the Kaplan–Meier method. The relationships of RAR with both RFS and OS were examined using univariate and multivariate Cox proportional hazards regression models. A nomogram was created using the identified independent prognostic factors from multivariate analysis to predict RFS. The discriminative ability and clinical usefulness of the nomogram were assessed by the concordance index (C-index), the calibration plots, time-dependent ROC analysis, and decision curve analysis (DCA).

**Results:**

Patients with higher preoperative RAR showed significantly poorer RFS and OS. Multivariate analysis identified high RAR as an independent prognostic factor for both RFS (HR: 1.731, 95% CI: 1.012 - 2.959) and OS (HR: 3.425, 95% CI: 1.196 - 9.806) in NMIBC patients. Based on these findings, RAR was incorporated into a nomogram model for predicting RFS. Compared to the baseline model without RAR, the new model exhibited an improved C-index (from 0.704 to 0.728). The calibration plots demonstrated excellent consistency of the nomogram-predicted probabilities for 1-, 3-, and 5-year RFS with the actual survival rates. The time-dependent ROC analysis revealed that the areas under the curve (AUC) values for RFS predictions at 1-, 3-, and 5- years were 0.806, 0.797 and 0.806. DCA validated that the nomogram yielded a superior net benefit within threshold probability ranges of 10% to 45% when compared to traditional staging systems.

**Conclusions:**

The findings suggest that preoperative RAR serves as a novel and independent prognostic factor for predicting RFS and OS in NMIBC cases.

## 1 Introduction

Bladder cancer represents the ninth most frequently diagnosed cancer worldwide and is the second most common urological neoplasm (1). At initial diagnosis, nearly 75% of bladder cancer cases are identified as non-muscle-invasive disease (NMIBC) (2). Transurethral resection of bladder tumor (TURBT) serves as the primary treatment for NMIBC and is typically followed by adjuvant intravesical therapy. Although the prognosis is relatively favorable, approximately 40%–80% of patients experience recurrence after initial treatment, and about 15% eventually progress to muscle-invasive bladder cancer (2–4). Moreover, among high-risk NMIBC patients, the progression rate to invasive disease can reach 50% or higher (5). In light of these clinical challenges, identifying effective prognostic biomarkers is crucial for early risk stratification and the development of individualized treatment strategies.

Currently, prognostic assessment of NMIBC primarily relies on risk stratification models developed by the European Organisation for Research and Treatment of Cancer (EORTC) (6) and the the Club Urológico Español de Tratamiento Oncológico (CUETO) (7). However, as these models are predominantly founded on clinicopathological parameters—such as tumor grade, stage, number, and size—they exhibit inherent limitations. In recent years, novel prognostic models incorporating molecular genetic features alongside clinicopathological factors (8) have demonstrated superior predictive performance. Nevertheless, due to the complexity and high cost of the required detection techniques, these models have not yet been widely adopted in routine clinical practice. Therefore, an urgent need exists to identify easily accessible, cost-effective, and reproducible biomarkers for accurate preoperative prediction of recurrence risk in NMIBC, which would significantly contribute to optimizing clinical management and improving patient outcomes.

Red cell distribution width (RDW) represents a standard component reported in complete blood cell tests. Emerging evidence indicates that elevated RDW, as a quantitative measure of erythrocyte volume heterogeneity, is significantly associated with systemic inflammation, heightened oxidative stress, and adverse prognosis in various cancers (9–12). Serum albumin (ALB) functions not only as a key indicator of nutritional status but also modulates the tumor microenvironment through its anti-inflammatory and immunoregulatory effects (13, 14). The RDW-to-ALB ratio (RAR) is a novel, integrated metric that offers a combined measure of overall inflammatory activity and nutritional condition. It has demonstrated significant prognostic value in malignancies such as hepatocellular carcinoma (15) and multiple myeloma (16). However, the prognostic role of RAR in NMIBC has not been established.

Accordingly, this research was designed to examine the relationship of preoperative RAR with both recurrence-free (RFS) and overall survival (OS) in individuals receiving TURBT for NMIBC, while also assessing its potential value as a new prognostic indicator.

## 2 Materials and methods

### 2.1 Study administration

**Figure 1:**
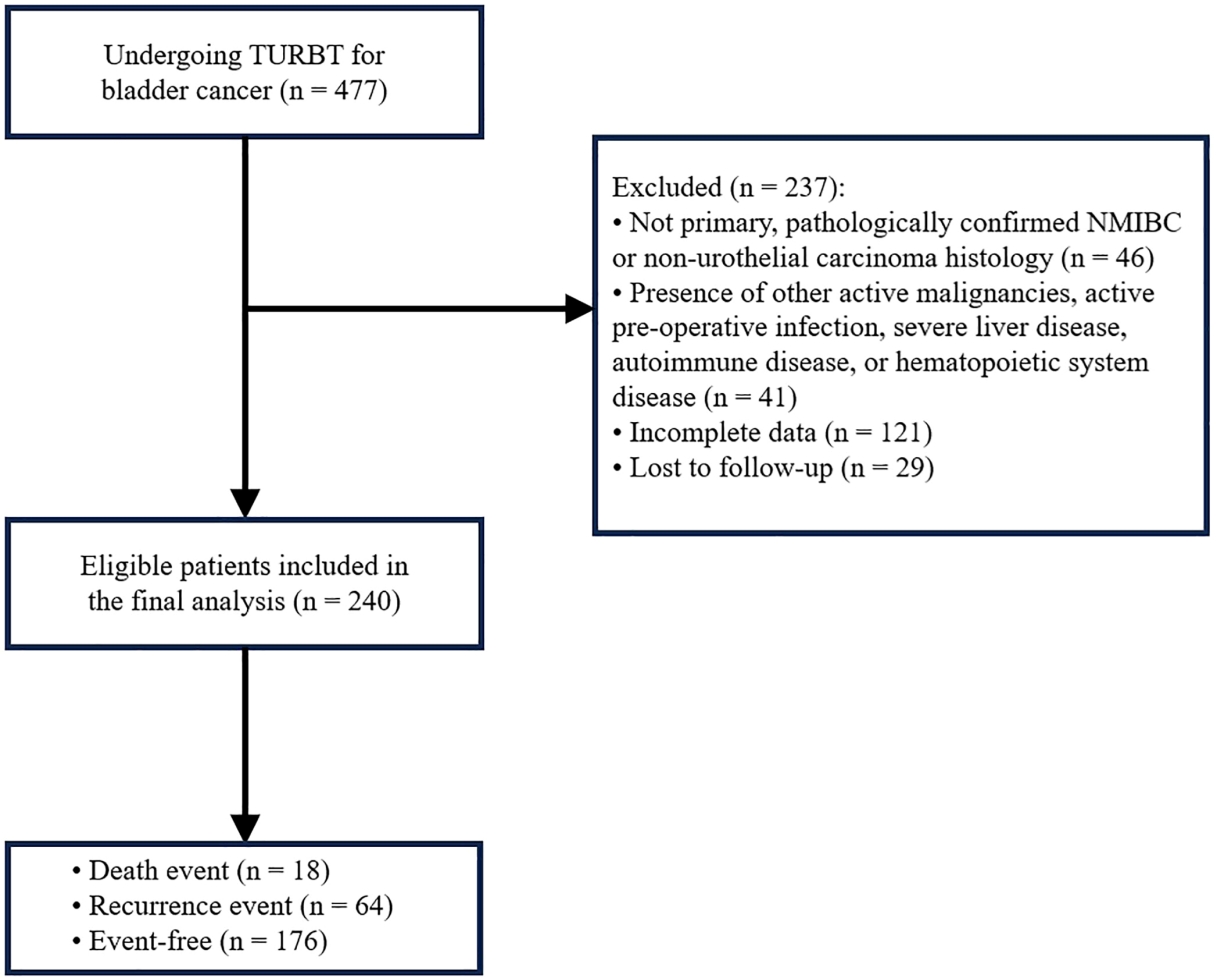
The flow chart for patient selection.

This retrospective study was conducted on 477 patients with bladder cancer who underwent TURBT at Shijiazhuang People’s Hospital from November 2013 to January 2024. Eligible patients met the following criteria: 1) histologically verified primary NMIBC; 2) underwent complete TURBT; 3) accessible and well-documented relevant data. Cases were excluded based on: 1) presence of distant metastasis or secondary bladder tumors; 2) preoperative active infection or severe liver diseases e.g., viral hepatitis, cirrhosis); 3) comorbid autoimmune or hematological disorders; 4) non-urothelial carcinoma histology; 5) missing key preoperative laboratory data (e.g., RDW, ALB) or incomplete clinicopathological information; 6) incomplete follow-up records or loss to follow-up; 7) concurrent active malignancies. Following the application of aforementioned criteria, a cohort of 240 patients were ultimately enrolled. Among them, 18 patients died, 64 experienced recurrence, and 176 remained event-free (**Figure 1**). The research protocol was reviewed and approved by the Ethics Committee of Shijiazhuang People’s Hospital. All procedures conducted in this research adhered to the ethical guidelines outlined in the Declaration of Helsinki.

### 2.2 Variables

Demographic characteristics, hematological parameters, and postoperative instillation therapy information were collected by retrospectively reviewing electronic medical records. Demographic data included gender, age, smoking history, history of abdominal surgery, and history of common chronic diseases. Hematological parameters comprised platelet-to-lymphocyte ratio (PLR), lactate dehydrogenase (LDH), neutrophil-to-lymphocyte ratio (NLR), ALB, RDW, and RAR. All hematological parameters were assessed using peripheral blood samples collected after fasting in the week preceding surgery, and analyzed using automated hematology analyzers in the hospital’s central laboratory. Postoperative pathological variables included tumor grade, tumor stage, tumor size, and tumor number. All pathological parameters were independently reevaluated by two blinded senior pathologists based on histological slides. Tumor grading was assessed using both the 2004 and 2016 World Health Organization (WHO) classifications, while tumor staging adhered to the American Joint Committee on Cancer (AJCC) TNM system (8th edition, 2017).

### 2.3 Follow-up and outcomes

All enrolled patients were monitored post-surgery under a structured surveillance schedule: assessments occurred every three months during the initial two years, semi-annually from year three to five, and yearly after that. Evaluations comprised urinalysis, urine cytology, urinary system ultrasound, and cystoscopy. The final follow-up date was March 2025. The primary endpoint was RFS, defined as the time from the date of TURBT to the first radiologically or cystoscopically confirmed intravesical recurrence, distant metastasis, or the last follow-up. The secondary endpoint was OS, calculated from the surgery date to death from any cause or the last follow-up.

### 2.4 Statistical analysis

The optimal cutoff values for RAR, PLR, LDH, NLR, ALB, and RDW were identified via receiver operating characteristic (ROC) analysis. According to the RAR threshold, subjects were stratified into groups with high and low RAR levels. Continuous variables were presented as median (interquartile range) and analyzed with the Mann–Whitney U test. Categorical variables were summarized as frequencies (percentages), with group comparisons performed using the chi-square test or Fisher’s test, as appropriate. Kaplan-Meier curves were plotted to illustrate survival, the log-rank test was used to compare differences. Univariate and multivariate Cox regression analyses were employed to evaluate factors independently associated with RFS and OS, reporting hazard ratios (HR) and 95% confidence intervals (CI). Prognostic factors identified as independent in the multivariate analysis (P < 0.05) were incorporated into a nomogram for estimating 1-, 3-, and 5-year RFS. The discriminative ability of the nomogram was assessed by the concordance index (C-index) and time-dependent ROC analysis. Calibration curves and bootstrap resampling (1000 repetitions) were used for internal validation to assess the nomogram’s calibration accuracy and stability. Decision curve analysis (DCA) was further conducted to quantify the clinical utility of the nomogram over a range of probability thresholds. The analyses were done in R (version 4.4.3). Statistical significance was defined as a two-sided P value < 0.05 for all tests.

## 3 Results

### 3.1 Patient features

The final analysis comprised 240 individuals diagnosed with NMIBC. Among them, 26 (10.83%) were female and 214 (89.17%) were male, with an overall median age of 69 years (interquartile range: 60–77). The median follow-up time was 58.21 months. ROC analysis indicated that RAR outperformed PLR, LDH, NLR, ALB, and RDW in predicting survival outcomes (**Supplementary Figure S1**). The optimal cutoff values for RAR, PLR, LDH, NLR, ALB, and RDW were determined to be 0.36, 135.71, 173.50, 3.22, 37.55, and 13.39, respectively (**Supplementary Table S1**). Based on the RAR cutoff, subjects were stratified into a high RAR cohort (RAR > 0.36, n = 59) and a low RAR cohort (RAR ≤ 0.36, n = 181). The baseline clinical characteristics and the survival outcomes for all patients are detailed in **Table 1** and **Supplementary Table S2**, respectively. Intergroup comparisons revealed no statistically notable disparities in gender, history of abdominal surgery, diabetes, coronary heart disease, smoking history, tumor number, tumor size, tumor grade, tumor stage, instillation therapy, LDH, NLR, or PLR (all P > 0.05). Nevertheless, the median age was significantly greater in the high RAR cohort relative to the low RAR cohort (P < 0.001). As the direct components of RAR, the high RAR cohort showed markedly reduced ALB along with elevated RDW levels, with statistical significance set at P < 0.001 for both comparisons. Additionally, the follow-up duration was markedly reduced in the high RAR cohort compared to the low RAR cohort (P = 0.043).

**Table 1:** Study characteristics.

Characteristic	Overall n = 240	Low RAR n = 181	High RAR n = 59	P value
Gender				0.438
Female	26 (10.83%)	18 (9.94%)	8 (13.56%)	
Male	214 (89.17%)	163 (90.06%)	51 (86.44%)	
Age, years	69.00 (60.00, 77.00)	66.00 (60.00, 74.00)	76.00 (68.00, 80.00)	<0.001
Smoking				0.113
No	163 (67.92%)	118 (65.19%)	45 (76.27%)	
Yes	77 (32.08%)	63 (34.81%)	14 (23.73%)	
Diabetes				0.878
No	206 (85.83%)	155 (85.64%)	51 (86.44%)	
Yes	34 (14.17%)	26 (14.36%)	8 (13.56%)	
Coronary heart disease				0.234
No	210 (87.50%)	161 (88.95%)	49 (83.05%)	
Yes	30 (12.50%)	20 (11.05%)	10 (16.95%)	
History of abdominal surgery				0.878
No	206 (85.83%)	155 (85.64%)	51 (86.44%)	
Yes	34 (14.17%)	26 (14.36%)	8 (13.56%)	
Number of tumors				0.838
Single	156 (65.00%)	117 (64.64%)	39 (66.10%)	
Multiple	84 (35.00%)	64 (35.36%)	20 (33.90%)	
Tumor size, cm	2.50 (2.00, 3.00)	2.50 (2.00, 3.00)	3.00 (2.00, 3.50)	0.109
Tumor grade				0.116
Low	139 (57.92%)	110 (60.77%)	29 (49.15%)	
High	101 (42.08%)	71 (39.23%)	30 (50.85%)	
Tumor stage				0.892
pTaN0M0	132 (55.00%)	100 (55.25%)	32 (54.24%)	
pT1N0M0	108 (45.00%)	81 (44.75%)	27 (45.76%)	
Instillation therapy				0.091
Immunotherapy	33 (13.75%)	21 (11.60%)	12 (20.34%)	
Chemotherapy	207 (86.25%)	160 (88.40%)	47 (79.66%)	
LDH, U/L				0.100
≤173.50	112 (46.67%)	79 (43.65%)	33 (55.93%)	
>173.50	128 (53.33%)	102 (56.35%)	26 (44.07%)	
NLR				0.083
≤3.22	197 (82.08%)	153 (84.53%)	44 (74.58%)	
>3.22	43 (17.92%)	28 (15.47%)	15 (25.42%)	
PLR				0.265
≤135.71	141 (58.75%)	110 (60.77%)	31 (52.54%)	
>135.71	99 (41.25%)	71 (39.23%)	28 (47.46%)	
ALB, g/L				<0.001
≤37.55	49 (20.42%)	8 (4.42%)	41 (69.49%)	
>37.55	191 (79.58%)	173 (95.58%)	18 (30.51%)	
RDW, %				<0.001
≤13.39	117 (48.75%)	109 (60.22%)	8 (13.56%)	
>13.39	123 (51.25%)	72 (39.78%)	51 (86.44%)	
Follow-up duration, months	58.21 (36.33, 98.80)	60.81 (42.44, 102.92)	51.51 (33.61, 86.79)	0.043

RAR, red blood cell distribution width-to-albumin ratio; LDH, lactate dehydrogenase; NLR, neutrophil-to-lymphocyte ratio; PLR, platelet-to-lymphocyte ratio; ALB, albumin; RDW, red blood cell distribution width. Continuous variables are expressed as median (interquartile range), while categorical variables are expressed as n (%).

### 3.2 RAR and survival outcomes

**Figure 2:**
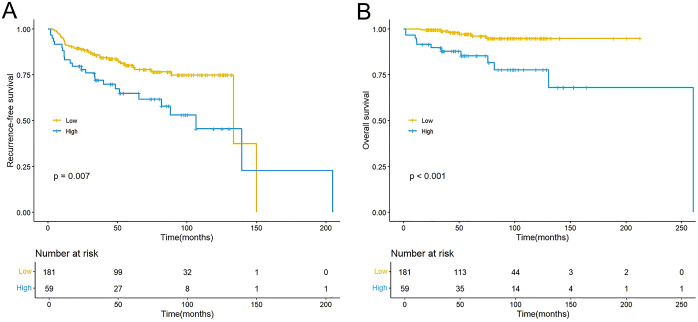
Kaplan–Meier curves for RFS **(A)** and OS **(B)** stratified by the RAR. RFS, recurrence-free survival; OS, overall survival; RAR, red blood cell distribution width-to-albumin ratio.

Kaplan-Meier analysis showed that the high RAR group exhibited inferior RFS (P = 0.007; **Figure 2A**) and OS (P < 0.001; **Figure 2B**). Univariate analysis revealed that elevated RAR served as a significant predictor for both reduced RFS (HR: 2.005, 95% CI: 1.195–3.363; P = 0.008; **Table 2**) and OS (HR: 5.540, 95% CI: 2.036–15.081; P = 0.001; **Table 3**). In a multivariate Cox regression model adjusted for potential confounders, a higher preoperative RAR level remained an independent predictor for both RFS (HR: 1.731, 95% CI: 1.012 - 2.959; P = 0.045; **Table 2**) and OS (HR: 3.425, 95% CI: 1.196 - 9.806; P = 0.022; **Table 3**). Furthermore, multivariate analysis confirmed that a history of abdominal surgery, larger tumor size, and T1 stage were independent predictors for RFS, while for OS, larger tumor size and elevated NLR were also identified as independent prognostic factors.

**Table 2:** Univariate and multivariate cox regression analysis for recurrence-free survival.

Characteristic	Univariate analysis		Multivariate analysis	
	HR (95% CI)	P value	HR (95% CI)	P value
Gender				
Female	Reference			
Male	2.505 (0.784 - 8.007)	0.121		
Age, years	1.030 (1.006 - 1.054)	0.012	1.012 (0.988 - 1.036)	0.344
Smoking				
No	Reference			
Yes	1.088 (0.645 - 1.837)	0.751		
Diabetes				
No	Reference			
Yes	1.021 (0.502 - 2.076)	0.953		
Coronary heart disease				
No	Reference			
Yes	1.629 (0.845 - 3.140)	0.145		
History of abdominal surgery				
No	Reference		Reference	
Yes	2.370 (1.276 - 4.404)	0.006	2.397 (1.281 - 4.484)	0.006
Tumor number				
Single	Reference			
Multiple	1.447 (0.875 - 2.393)	0.15		
Tumor size, cm	1.498 (1.248 - 1.799)	<0.001	1.474 (1.236 - 1.758)	<0.001
Tumor grade				
Low	Reference			
High	1.635 (0.989 - 2.701)	0.055		
Tumor stage				
pTaN0M0	Reference		Reference	
pT1N0M0	2.189 (1.295 - 3.700)	0.003	2.003 (1.156 - 3.468)	0.013
Instillation therapy				
Immunotherapy	Reference		Reference	
Chemotherapy	0.535 (0.293 - 0.974)	0.041	0.684 (0.360 - 1.298)	0.245
LDH, U/L				
≤173.50	Reference			
>173.50	0.839 (0.501 - 1.406)	0.504		
NLR				
≤3.22	Reference			
>3.22	1.554 (0.876 - 2.757)	0.132		
PLR				
≤135.71	Reference			
>135.71	1.421 (0.861 - 2.343)	0.169		
RAR				
≤0.36	Reference		Reference	
>0.36	2.005 (1.195 - 3.363)	0.008	1.731 (1.012 - 2.959)	0.045

HR, hazard ratio; CI, confidence interval; LDH, lactate dehydrogenase; NLR, neutrophil-to-lymphocyte ratio; PLR, platelet-to-lymphocyte ratio; RAR, red blood cell distribution width-to-albumin ratio.

**Table 3:** Univariate and multivariate cox regression analysis for overall survival.

Characteristic	Univariate analysis		Multivariate analysis	
	HR (95% CI)	P value	HR (95% CI)	P value
Gender				
Female	Reference			
Male	0.947 (0.216 - 4.147)	0.942		
Age, years	1.095 (1.036 - 1.157)	0.001	1.053 (0.987 - 1.124)	0.120
Smoking				
No	Reference			
Yes	0.609 (0.199 - 1.870)	0.386		
Diabetes				
No	Reference			
Yes	1.901 (0.619 - 5.842)	0.262		
Coronary heart disease				
No	Reference			
Yes	2.109 (0.686 - 6.490)	0.193		
History of abdominal surgery				
No	Reference			
Yes	0.456 (0.060 - 3.452)	0.447		
Tumor number				
Single	Reference			
Multiple	0.988 (0.365 - 2.673)	0.981		
Tumor size, cm	1.821 (1.349 - 2.459)	<0.001	1.494 (1.105 - 2.021)	0.009
Tumor grade				
Low	Reference		Reference	
High	4.411 (1.435 - 13.559)	0.01	1.857 (0.552 - 6.250)	0.318
Tumor stage				
pTaN0M0	Reference			
pT1N0M0	2.464 (0.862 - 7.043)	0.093		
Instillation therapy				
Immunotherapy	Reference		Reference	
Chemotherapy	0.331 (0.122 - 0.902)	0.031	0.531 (0.187 - 1.511)	0.235
LDH, U/L				
≤173.50	Reference			
>173.50	0.747 (0.282 - 1.980)	0.558		
NLR				
≤3.22	Reference		Reference	
>3.22	6.577 (2.498 - 17.313)	<0.001	3.714 (1.298 - 10.629)	0.014
PLR				
≤135.71	Reference			
>135.71	2.247 (0.854 - 5.912)	0.101		
RAR				
≤0.36	Reference		Reference	
>0.36	5.540 (2.036 - 15.081)	0.001	3.425 (1.196 - 9.806)	0.022

HR, hazard ratio; CI, confidence interval; LDH, lactate dehydrogenase; NLR, neutrophil-to-lymphocyte ratio; PLR, platelet-to-lymphocyte ratio; RAR, red blood cell distribution width-to-albumin ratio.

### 3.3 Nomogram development and evaluation

**Figure 3:**
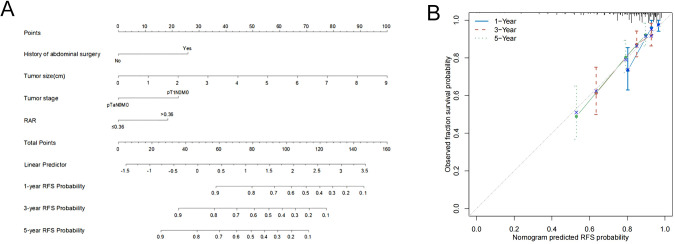
Nomograms and calibration curves for the prediction of 1-, 3- and 5-year RFS. Nomograms for 1-, 3- and 5-year RFS **(A)** prediction. Calibration curves for estimating the prediction of 1-, 3- and 5-year RFS **(B)** between the prediction and the actual observation. RFS, recurrence-free survival; RAR, red blood cell distribution width-to-albumin ratio.

**Figure 4:**
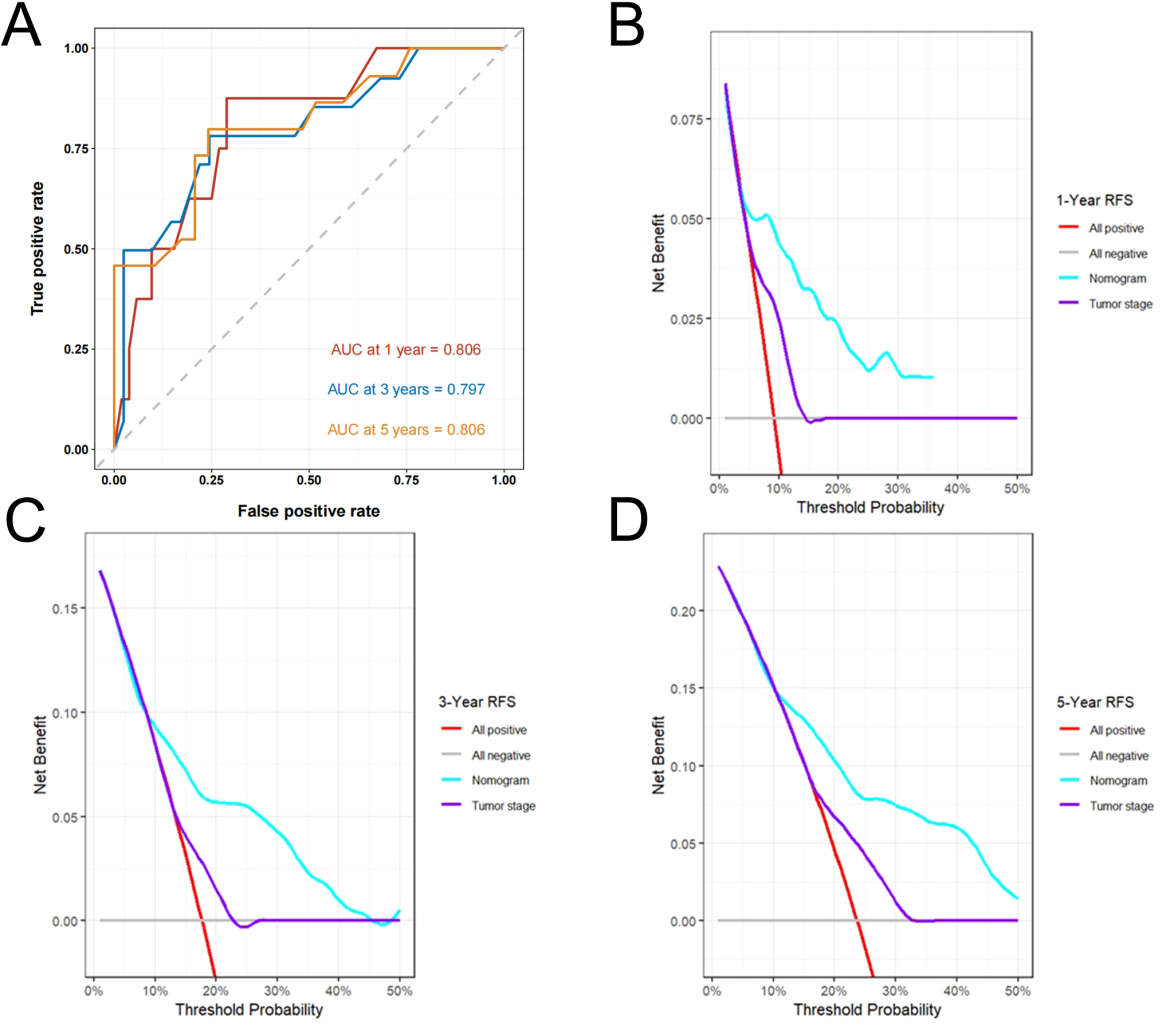
ROC curves and decision curve analyses of the nomogram for RFS prediction. ROC curves for RFS **(A)**. Decision curve analyses for 1-year **(B)**, 3-year **(C)** and 5-year **(D)** RFS prediction. ROC, receiver operating characteristic; RFS, recurrence-free survival.

Multivariate Cox regression identified four independent predictors—history of abdominal surgery, tumor size, tumor stage, and RAR—which were incorporated into the development of a nomogram designed to estimate 1-, 3-, and 5-year RFS (**Figure 3A**). The calibration plots, generated using 1000 bootstrap resamples, demonstrated excellent consistency between model-predicted and actual RFS at 1-, 3-, and 5-year (**Figure 3B**). A C-index of 0.728 was demonstrated by the nomogram, indicating strong predictive performance (**Table 4**). This value surpassed the baseline model that excluded RAR (C-index = 0.704), suggesting that the inclusion of RAR significantly enhanced the model’s discriminative ability. After internal validation with 1000 bootstrap repetitions, the calibrated C-index was 0.715, further confirming the model’s robustness. The area under the curve (AUC) values of 0.806, 0.797, and 0.806 were shown to be indicative of 1-, 3-, and 5-year RFS, respectively, as demonstrated by time-dependent ROC analysis (**Figure 4A**), confirming the nomogram’s consistent and excellent predictive accuracy across different time points. Furthermore, DCA showed that over a broad spectrum of threshold probabilities (10%–45%), the clinical net benefit obtained by applying this nomogram was consistently higher than the strategies of “treating all patients” or “treating none,” and also superior to the strategy based solely on tumor stage (**Figures 4B–D**).

**Table 4:** C-Index of the nomogram for the prediction of survival outcomes.

Outcome	Models	C-index	Optimism	95%CI	Corrected C-index	ΔC-index
RFS	Nomogram without RAR	0.704	0.009	(0.629, 0.778)	0.695	
	Nomogram*	0.728	0.013	(0.655, 0.801)	0.715	
	Nomogram without RAR vs Nomogram					0.024

*History of abdominal surgery, tumor size, tumor stage, RAR were included in the nomogram for recurrence-free survival prediction. RFS, recurrence-free survival; RAR, red blood cell distribution width-to-albumin ratio; CI, confidence interval.

## 4 Discussion

Through a systematic analysis, this research provides the initial evidence that preoperative RAR represents an independent and novel predictor of postoperative outcomes for NMIBC patients undergoing TURBT. Kaplan–Meier analysis demonstrated that the high RAR cohort exhibited markedly shorter RFS and OS. Importantly, following adjustment for covariates in the multivariate Cox regression analyses, a higher RAR level was significantly associated with both reduced RFS (HR: 1.731) and OS (HR: 3.425). This finding suggests that RAR provides complementary prognostic information to established clinicopathological factors (e.g., tumor size and stage), offering an efficient and easily accessible biomarker for risk stratification in NMIBC management.

RAR demonstrated superior prognostic accuracy over established markers including LDH, NLR, PLR, ALB, and RDW. This enhancement in predictive utility is likely attributable to the index’s composite nature, which captures both inflammatory and nutritional pathophysiological processes. RDW, a key parameter reflecting erythrocyte volume heterogeneity, is elevated not only in anemias but also closely associated with chronic inflammatory states, oxidative stress, and microenvironment dysregulation (17, 18). Research has demonstrated that inflammatory cytokines (e.g., IL-6, TNF-α) can inhibit erythrocyte maturation, leading to increased RDW (19), while simultaneously activating oncogenic pathways such as STAT3, promoting tumorigenesis, immune evasion, and driving bladder cancer progression (20). Research by Fukuoka et al. (21) further confirmed that elevated RDW was correlated with shorter RFS in NMIBC patients. In contras, hypoalbuminemia serves as a marker of systemic inflammation and malnutrition (22, 23). It may facilitate tumor development and progression by impairing immune surveillance, promoting angiogenesis, and supporting aberrant cell proliferation (24). A previous investigation by Shen et al. (25) also identified that low preoperative albumin level is an independent predictor of poorer OS in individuals diagnosed with urothelial carcinoma of the bladder and treated with TURBT. The RAR index proposed in this study integrates both RDW and albumin, simultaneously reflecting two critical pathophysiological processes—systemic inflammation and nutritional deficiency—and demonstrates enhanced prognostic capability for survival relative to each parameter individually.

It is noteworthy that the median age at baseline was markedly greater in the high RAR cohort compared to the low RAR cohort. On one hand, RDW tends to increase with age, potentially due to age-related changes in hematopoiesis and chronic inflammatory states (“inflammaging”) (26–28). On the other hand, ALB levels often slightly decline with aging (29). Therefore, the observed increase in RAR with age is expected, indicating that this index may effectively capture age-related physiological vulnerability, which is associated with poorer cancer outcomes. Additionally, the shorter median follow-up time in the high RAR group likely reflects earlier occurrence of endpoint events (e.g., recurrence, progression, or death) in these patients (30), indirectly supporting the adverse predictive value of RAR. It should be emphasized that the Kaplan-Meier plots and Cox regression models used in this study effectively account for such differences in follow-up duration, ensuring the reliability of comparative results. Most importantly, following adjustment for possible confounders in the multivariate analysis, RAR retained its independent prognostic significance, indicating that it reflects systemic inflammatory and nutritional status beyond the effect of age alone.

To facilitate clinical translation, we developed and validated a RFS-predicting nomogram incorporating RAR and demonstrated its predictive accuracy and clinical utility. Furthermore, this study innovatively identified a “history of abdominal surgery” as an independent predictor of tumor recurrence. Although the underlying mechanisms remain unclear, it is hypothesized that anatomical alterations, chronic inflammation, and immune microenvironment changes resulting from previous surgeries may contribute to this association (31, 32). Although RAR was also established to be an independent factor in predicting OS, a predictive model for OS was not constructed due to the limited number of OS events, in order to prevent overfitting and ensure model stability. Despite extensive research on various prognostic markers for NMIBC, urinary cytology remains the only biomarker currently recommended by professional guidelines for NMIBC surveillance. However, its sensitivity is limited, and no biomarker has been endorsed for routine follow-up management of NMIBC (33, 34). In this context, RAR—as a novel, economical, and readily accessible blood-based index—represents a promising adjunct tool for enhancing risk stratification and prognostic evaluation in NMIBC.

This study has several limitations. First, as a retrospective study conducted at a single institution, the findings may be susceptible to selection bias. Second, the sample size was relatively small. This limited the statistical power for some analyses, most notably the multivariate analysis for OS, and the results for OS should be interpreted with caution. Future studies with larger cohorts are needed to corroborate these findings. Third, the optimal cutoff value for RAR requires external validation in larger, multicenter prospective cohorts. Finally, although known confounding factors were adjusted for, unmeasured variables (such as detailed comorbidities) may still influence the results.

## 5 Conclusion

In summary, this study demonstrates that preoperative RAR is an independent prognostic indicator in NMIBC patients following TURBT. Elevated RAR is significantly related to reduced RFS and OS. Furthermore, the RFS-predicting nomogram incorporating RAR significantly improves the accuracy of individualized recurrence risk assessment, thereby assisting clinicians in refined risk stratification and informed treatment decision-making, with the best possible chance of enhancing patient outcomes. Future research directions will focus on advancing external validation of this model, elucidating the mechanistic underpinnings of RAR, and progressively establishing a multi-marker quantitative prediction system to enable more efficient clinical translation and application.

## Data Availability

The original contributions presented in the study are included in the article/**Supplementary Material**. Further inquiries can be directed to the corresponding author.
