# Supplementary material for: Association between the red cell distribution width-to-albumin ratio and recurrence-free survival and overall survival in patients with non-muscle-invasive bladder cancer: a retrospective cohort study
Source: Front Oncol. 2026 Jan 5;15:1710047. doi: 10.3389/fonc.2025.1710047 (PMC12812645; doi:10.3389/fonc.2025.1710047)
Supplement: Supplementary file 1 [file DataSheet1.docx]

**Figure S1 ROC curves for the determination of the optimal cutoff level of RAR, LDH, NLR, PLR, albumin and RDW.**


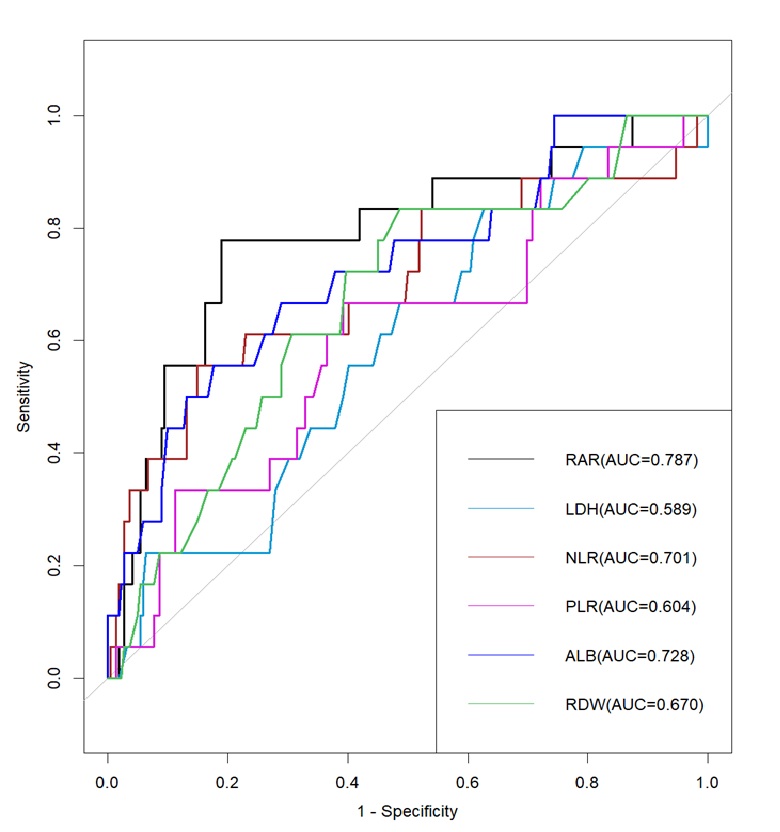


**Abbreviations:** ROC, receiver operating characteristic; RAR, red blood cell distribution width-to-albumin ratio; LDH, lactate dehydrogenase; NLR, neutrophil-to-lymphocyte ratio; PLR, platelet-to-lymphocyte ratio; ALB, albumin; RDW, red blood cell distribution width; AUC: area under the curve.

| Variables | Cut-off value | Sensitivity | Specificity | Youden Index | AUC |
| --- | --- | --- | --- | --- | --- |
| RAR | 0.36 | 0.78 | 0.81 | 0.59 | 0.787 |
| LDH | 173.50 | 0.83 | 0.37 | 0.20 | 0.589 |
| NLR | 3.22 | 0.56 | 0.85 | 0.41 | 0.701 |
| PLR | 135.71 | 0.67 | 0.61 | 0.28 | 0.604 |
| ALB | 37.55 | 0.56 | 0.82 | 0.38 | 0.728 |
| RDW | 13.39 | 0.83 | 0.51 | 0.34 | 0.670 |

**Table S1 Results from receiver operating characteristic analysis.**

**Abbreviations:** RAR, red blood cell distribution width-to-albumin ratio; LDH, lactate dehydrogenase; NLR, neutrophil-to-lymphocyte ratio; PLR, platelet-to-lymphocyte ratio; ALB, albumin; RDW, red blood cell distribution width; AUC: area under the curve.

| **Characteristic** | **Overall N = 240** | **Low RAR N = 181** | **High RAR N = 59** | **P value** |
| --- | --- | --- | --- | --- |
|  |  |  |  |  |
| OS status |  |  |  | <0.001 |
| Alive / Censored | 222 (92.50%) | 177 (97.79%) | 45 (76.27%) |  |
| Death | 18 (7.50%) | 4 (2.21%) | 14 (23.73%) |  |
| RFS status |  |  |  | 0.002 |
| Censored | 176 (73.33%) | 142 (78.45%) | 34 (57.63%) |  |
| Recurrence | 64 (26.67%) | 39 (21.55%) | 25 (42.37%) |  |

**Table S2 Survival outcomes stratified by preoperative RAR.**

**Abbreviations**: OS, overall survival; RFS, recurrence-free survival; RAR, red blood cell distribution width-to-albumin ratio.

Continuous variables are expressed as median (interquartile range), while categorical variables are expressed as n (%).

Note:​ Censored data include patients who were event-free at the end of the study.
